# Assessing the Insecticidal Performance of *Commiphora myrrha* Essential Oil Against *Prostephanus truncatus* and *Sitophilus zeamais* Using a Metabolomic Approach

**DOI:** 10.3390/plants14193031

**Published:** 2025-09-30

**Authors:** Nickolas G. Kavallieratos, Maria C. Boukouvala, Constantin S. Filintas, Demeter Lorentha S. Gidari, Anna Skourti, Vasiliki Panagiota C. Kyrpislidi, Filippo Maggi, Riccardo Petrelli, Eleonora Spinozzi, Marta Ferrati, Cristina Teruzzi, Fabrizio Araniti

**Affiliations:** 1 Laboratory of Agricultural Zoology and Entomology, Department of Crop Science, Agricultural University of Athens, 75 Iera Odos str., 11855 Athens, Greece; mbouk@aua.gr (M.C.B.); kfilintas@aua.gr (C.S.F.); dlgidari@aua.gr (D.L.S.G.); annaskourti@aua.gr (A.S.); stud4419112@aua.gr (V.P.C.K.); 2 Chemistry Interdisciplinary Project (ChIP) Research Center, School of Pharmacy, University of Camerino, Via Madonna delle Carceri, 62032 Camerino, Italy; filippo.maggi@unicam.it (F.M.); riccardo.petrelli@unicam.it (R.P.); eleonora.spinozzi@unicam.it (E.S.); marta.ferrati@unicam.it (M.F.); 3 Department of Agricultural and Environmental Sciences-Production, Landscape, Agroenergy, University of Milan, 20133 Milan, Italy; cristina.teruzzi@unimi.it (C.T.); fabrizio.araniti@unimi.it (F.A.)

**Keywords:** larger grain borer, maize weevil, stored-product pests, natural product, metabolome, GC-MS, HPLC

## Abstract

Botanical insecticides have gained interest due to a rising demand for environmentally friendly pest control methods for stored-product protection. The insecticidal effectiveness of the essential oil (EO) obtained from the oleo-gum-resin of myrrh (*Commiphora myrrha* (Nees) Engl.), against *Prostephanus truncatus* (Horn) and *Sitophilus zeamais* Motschulsky, and the metabolic shifts of the two species, were investigated in this work. A thorough gas chromatography-mass spectrometry (GC-MS) investigation showed that the composition of this EO was dominated by furanosesquiterpenes, specifically, furanoeudesma-1,3-diene and curzerene. *Commiphora myrrha* EO treatments, especially at 1000 ppm, resulted in high adult mortality for *P. truncatus* (up to 85.6%), while *S. zeamais* showed only moderate mortality (up to 25.6%). To investigate the different species-specific effectiveness of the EO, untargeted GC-MS metabolomic profiling was conducted to elucidate the impact of the EO on the metabolism of the insects, with subsequent data analysis employing multivariate, univariate, and network methods. Each species reacts differently to the treatments (myrrh EO versus the synthetic insecticide pirimiphos-methyl (PM)), according to the analysis results. In particular, myrrh EO caused distinct shifts in metabolic pathways that varied between *P. truncatus* and *S. zeamais*. Overall, *C. myrrha* EO exhibits potential as a botanical insecticide, especially against *P. truncatus*, and it causes metabolic disturbances specific to the species. The results demonstrate the significance of metabolomic technologies in assessing bioinsecticide mechanisms and lend credence to their possible incorporation in integrated pest management methodologies or their contribution to the creation of diagnostic indicators of insecticidal exposure.

## 1. Introduction

The genus *Commiphora* Jacq. includes more than 150 plant species, which provide vital ecosystem services in their native habitats, supporting local wildlife biodiversity [1,2]. *Commiphora myrrha* (Nees) Engl. (Burseraceae) is a spiny shrub with prominent thorns that is adapted to arid regions characterized by low rainfall and shallow rocky soils [1,3,4]. It occurs mainly in northeast Africa (Somalia and Kenya) and as far as parts of the Arabian Peninsula [5]. This plant species produces short, dioecious yellow–red flowers arranged in a panicle inflorescence, which later develop into small, egg-shaped brown fruits [6]. Oleo-gum-resin obtained from *Commiphora* species has a long history of use for medicinal and cultural practices [7,8,9]. Ancient civilizations across Africa and Eurasia employed it to treat fever, gastrointestinal illnesses, wounds, pain, joint inflammation, bone fractures, skin conditions, and metabolic disorders [10,11,12,13,14]. Extracts from *C. myrrha* exhibited anti-inflammatory, antimicrobial, antiseptic, analgesic, anti-cancer, and anti-venom properties [15,16,17]. Consequently, myrrh oleo-gum-resin is a valuable resource for applications in cosmetic, pharmaceutical, and food industries [8,18].

The myrrh oleo-gum-resin has attracted considerable scientific attention due to the rich content of bioactive compounds [19,20,21,22,23], being characterized by a water-soluble gum, an alcohol-soluble resin, and a volatile fraction (essential oil, EO) mainly constituted by furanosesquiterpenes [24,25]. Previous reports have noted the insecticidal properties of *C. myrrha* against various species, such as *Aedes aegypti* (L.) (90.0% larval mortality after 24 h at 1000 ppm of *C. myrrha* resin), *Ae. albopictus* (Skuse), *Anopheles gambiae* Giles, and *An. stephensi* Liston (Diptera: Culicidae) (high larvicidal potential for all species; LC_50_ from 4.42 to 16.81) and stored-product insects including *Trogoderma granarium* Everts (Coleoptera: Dermestidae) (73.3% larval repellency after 72 h at 4 μg/cm^2^ grain), *Sitophilus oryzae* (L.) (Coleoptera: Curculionidae) (43.3% adult mortality after seven days at 728 ppm wheat) and two tenebrionids *Tenebrio molitor* L. (86.7% larval mortality after seven days at 1000 ppm wheat) and *Alphitobius diaperinus* (Panzer) (91.1% larval mortality after seven days at 1000 ppm wheat) [23,26,27,28,29].

Insect species commonly found in storage units cause considerable losses to the global food industry, even threatening public health [30,31]. Primary pests such as *Prostephanus truncatus* (Horn) (Coleoptera: Bostrychidae) and *Sitophilus zeamais* Motschulsky (Coleoptera: Curculionidae) are major threats to stored grain products worldwide. A large part of their life cycle takes place inside kernels, rendering their detection and management efforts complex and costly. Their control strategies mainly focus on the population suppression, using chemical (e.g., fumigation, diatomaceous earths) or biological insecticides, e.g., plant-based insecticidal agents [32,33].

Metabolomics is a fast-evolving technique that entails a comprehensive examination of all low-molecular-weight metabolites within a particular organism or cell. Identification of probable biochemical pathways linked to different physiological processes is made possible by the capacity to profile metabolites under certain stresses or under different growth conditions [34,35]. Investigating the metabolic profiles of *Callosobruchus chinensis* (L.) (Coleoptera: Bruchidae) larvae subjected to 2% O_2_ + 18% CO_2_, both *v*/*v*, Cui et al. [35] demonstrated that intricate environmental stressors have an impact on the metabolism of this species. Moreover, Ferri et al. [36] revealed the association between the rearing media and insect metabolism by evaluating the metabolomic profile of *Tenebrio molitor* L. (Coleoptera: Tenebrionidae) larvae when cultured on different rearing media. The metabolomic analysis of adults and larvae of two tenebrionids (*A. diaperinus* and *T. molitor*), treated with *C. myrrha* EO, revealed both species- and stage-specific susceptibility to the tested insecticidal agent [28].

The limited knowledge on the insecticidal properties of *C. myrrha* EO [23,26,27,28,29], as well as the outcomes of the first try on the observation of the metabolic disruptions on the *C. myrrha* EO-treated *A. diaperinus* and *T. molitor* [28], led us to perform an in-depth evaluation of the pesticidal activity of *C. myrrha* EO on *S. zeamais* and *P. truncatus* adults, including a metabolomic analysis to assess the possible mode of action.

## 2. Results

### 2.1. Chemical Analysis of the Essential Oil (EO)

The characterization of *C. myrrha* EO is in line with that of Kavallieratos et al. [28]. The GC-MS analysis of *C. myrrha* EO led to the identification of 97.8% of the total composition. The predominant compounds were furanosesquiterpenes (86.1%), while sesquiterpene hydrocarbons (9.2%), esters (1.5%), and oxygenated sesquiterpenes (0.9%) gave a minor contribution. Furanoeudesma-1,3-diene was the most abundant compound (40.2%), followed by curzerene (26.1%) and lindestrene (12.9%), while (*R*)(5*E*,9*E*)-8-methoxy-3,6,10-trimethyl-4,7,8,11-tetrahydrocyclodeca[b]furan (4.6%), isofuranodiene (1.8%), atractylone (0.4%), and furanoeudesma-1,4-diene (0.3%) were found in minor percentages. The main sesquiterpene hydrocarbons were β-elemene (3.4%) and germacrene B (1.8%). The HPLC-DAD analysis was conducted to avoid the thermal degradation of furanosesquiterpenes (Cope-rearrangement) and confirmed the predominance of furanoeudesma-1,3-diene (67.3 g/100 g EO), curzerene (18.4 g/100 g EO), and isofuranodiene (6.2 g/100 g EO).

### 2.2. Main Effects and Interactions

The data analysis indicated that exposure was significant across both species. Concerning all main effects between exposures, concentration and formulation, along with their respective interaction, were significant for both species. Within exposure, the main effects/interaction were significant for *S. zeamais* and *P. truncatus* adults (Table 1).

### 2.3. Efficacy of C. myrra EO Against S. zeamais Adults

At 500 ppm, the EO showed no lethal effect against *S. zeamais* adults for the first six days, while at 1000 ppm, no mortality data were recorded until day five. On the final day, mortality reached 10.0 and 25.6% at 500 and 1000 ppm, respectively. In contrast, PM demonstrated a steady increase in mortality throughout the exposure period, ultimately achieving complete mortality by the end of the treatments. The *C. myrra* EO and the PM significantly differed among all the exposure intervals except for the 16 h interval at 500 ppm. Within each treatment, (*C. myrra* EO and PM) and concentration (500 and 1000 ppm), there were significant differences among the exposure intervals (Table 2).

### 2.4. Efficacy of C. myrra EO Against P. truncatus Adults

The mortality of *P. truncatus* adults varied significantly between treatments, with the EO displaying a less pronounced effect compared to PM. Specifically, at 500 ppm, EO treatments resulted in minimal mortality during the first four days, peaking at 30% by the fifth day and gradually increasing to 48.9% by the last day. At the highest dose (1000 ppm), the results indicated elevated efficacy, with mortality steadily rising from day two, reaching a high level (85.6%) by the end of the treatments. The PM treatment induced mortality, which began to significantly increase by the first day of exposure, with 16.7% at 500 and 1000 ppm of the EO. On the fifth day, recorded mortality exceeded 77% and almost complete mortality was achieved by day 7 (Table 3).

### 2.5. GC-MS-Driven Untargeted Metabolomic Analysis

**Figure 1:**
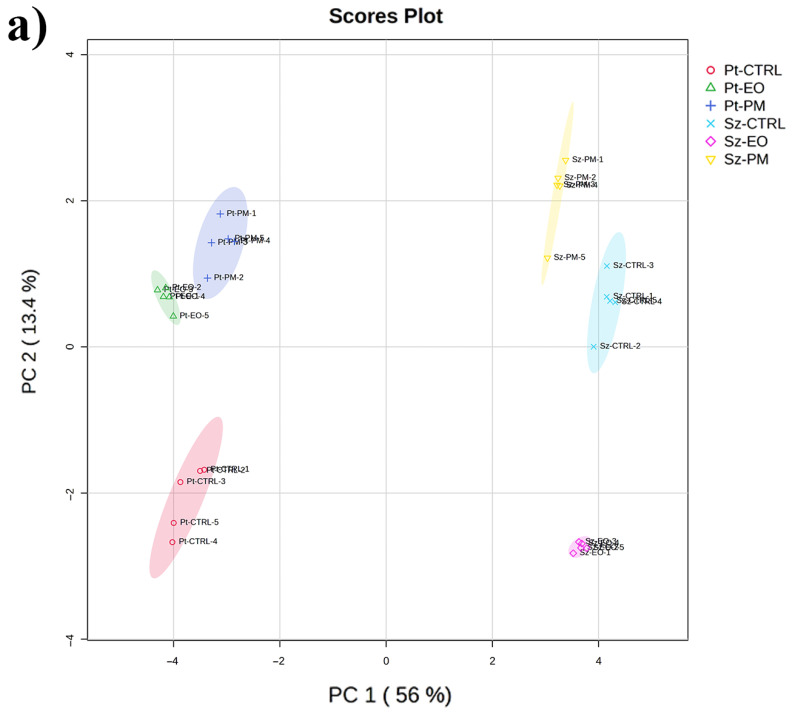

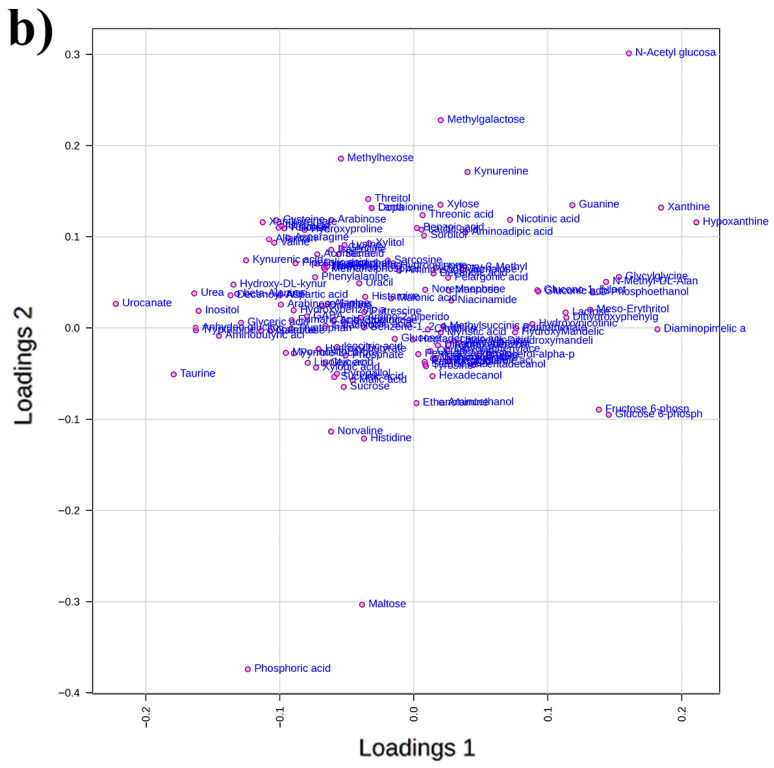
Multivariate principal component analysis (PCA) of the metabolic profiles of the adult stage of *Prostephanus truncatus* (Pt) and *Sitophilus zeamais* (Sz) treated with *Commiphora myrrha* essential oil (EO), the insecticide pirimiphos-methyl (PM) and control (CTRL). (**a**) PCA scores plot displaying the separation of metabolic profiles across the six experimental groups. (**b**) The PCA loadings plot illustrates the contribution of individual metabolites to the separation observed in the scores plot. Metabolites further from the origin have a stronger impact on group differentiation. N = 5.

The PCA revealed that species identity dominated variation, with PC1 explaining 56% of the total variance and clearly segregating *P. truncatus* (negative PC1 values) from *S. zeamais* (positive PC1 values), while PC2 (13.4% of variance) captured treatment effects within each species, placing untreated *P. truncatus* at low PC2 values and untreated *S. zeamais* at moderately higher values, shifting *P. truncatus* slightly upward and *S. zeamais* sharply downward under *C. myrrha* EO exposure and moving both species toward positive PC2 under organophosphate (PM) treatment, with a larger shift in *S. zeamais* (Figure 1a). The loading plot showed that metabolites with strongly positive PC1 loadings comprised sugar derivatives and nucleotide bases, N-acetylglucosamine, xanthine, hypoxanthine, glucose-6-phosphate, fructose-6-phosphate, whereas small organic acids and certain amino acids such as taurine, urocanate and phosphoric acid carried strongly negative PC1 loadings; PC2 separated methylated or unusual carbohydrates (methylgalactose, methylhexose, threitol) with positive loadings from canonical sugar phosphates and disaccharides (maltose, phosphoric acid) with negative loadings, with a central cluster of moderate-loading amino acids, organic acids and simple sugars lying between these extremes (Figure 1b, Table S1).

We used PCA on the entire dataset to develop an unbiased overview of its structure, which highlighted differences between species and the effects of our treatments. Next, we carried out PLS-DA on a single species to focus more sharply on separating the treatment groups. This supervised method should reveal detailed, species-specific metabolic changes and build on the broader patterns identified by the PCA.

**Figure 2:**
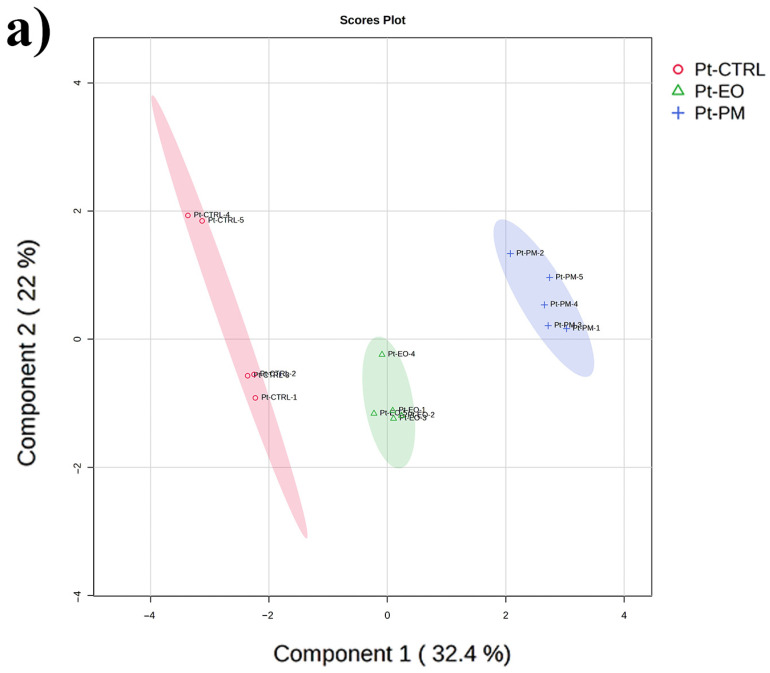

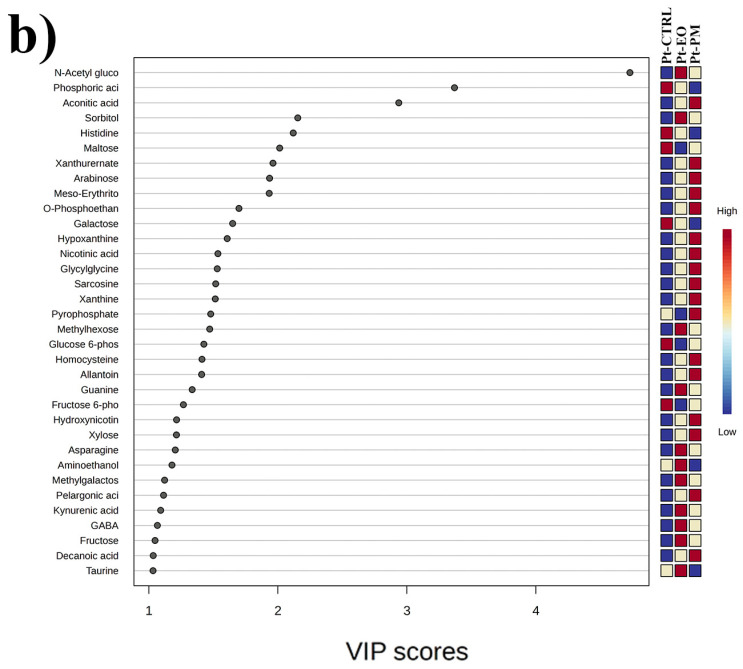

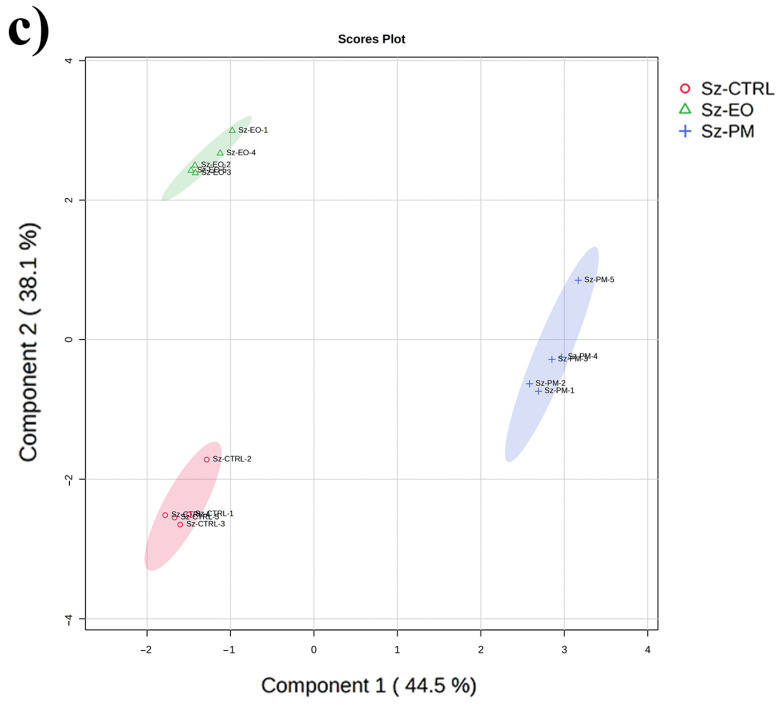

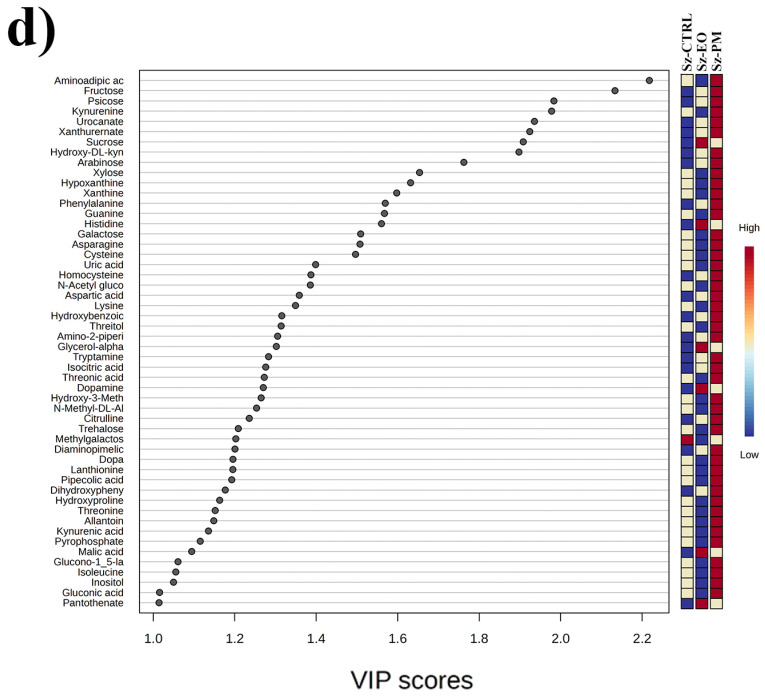
Multivariate partial least square discriminant analysis (PLS-DA) of adult stage of *Prostephanus truncatus* (Pt) and *Sitophilus zeamais* (Sz) treated with *Commiphora myrrha* essential oil (EO), the insecticide pirimiphos-methyl (PM) and control (CTRL). (**a**) *Prostephanus truncatus* PLS-DA scores plot displaying the separation of metabolic profiles across the different experimental groups. (**b**) PLS-DA VIP scores built on *Prostephanus truncatus* data illustrate individual metabolites’ contribution to the separation observed in the scores plot. (**c**) *Sitophilus zeamais* PLS-DA scores plot displaying the separation of metabolic profiles across the six experimental groups. (**d**) PLS-DA VIP scores built on *Sitophilus zeamais* data illustrate individual metabolites’ contribution to the separation observed in the scores plot. N = 5.

When PLS-DA was applied solely to *P. truncatus*, LV1 distinguished untreated controls at the origin from EO-exposed insects displaced positively and PM-treated insects displaced negatively, and LV2 further separated EO specimens (positive LV2) from PM specimens (negative LV2), with cross-validation yielding high R^2^ and Q^2^ and permutation tests confirming significance (Figure 2a, Table S1). VIP scores greater than 1 identified 36 metabolites driving class separation, including sugar and polyol intermediates, phosphorylated compounds, amino acids and derivatives, nucleotide-related molecules and organic acids plus N-acetylglucosamine and homocysteine (Figure 2b, Table S1). In *S. zeamais*, PLS-DA axis 1 captured the largest EO-induced shift away from controls and PM-treated insects, and axis 2 separated PM from controls, with robust cross-validation and permutation-test results (Figure 2c), while VIP ≥ 1 selection yielded 53 key metabolites spanning carbohydrate metabolism, amino acids and derivatives, nucleobase turnover, organic acids, the tryptophan–kynurenine axis, and cofactors or aminosugars that collectively define distinct EO versus PM metabolic signatures in each species (Figure 2d, Table S1).

**Figure 3:**
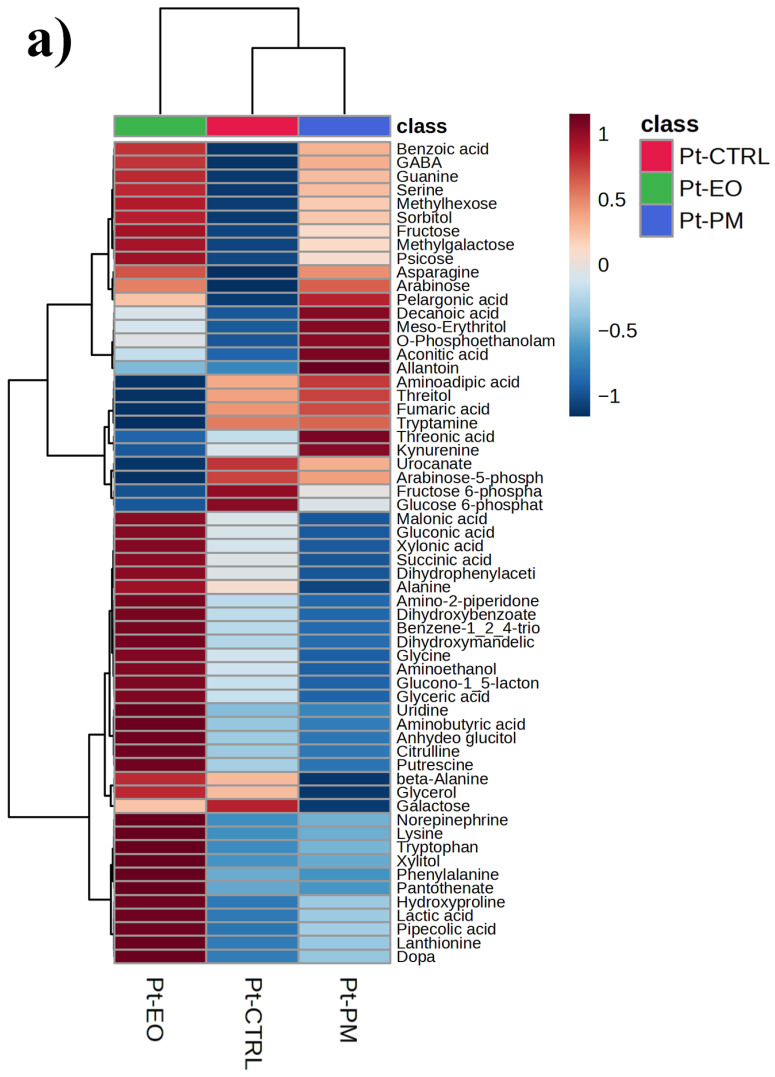

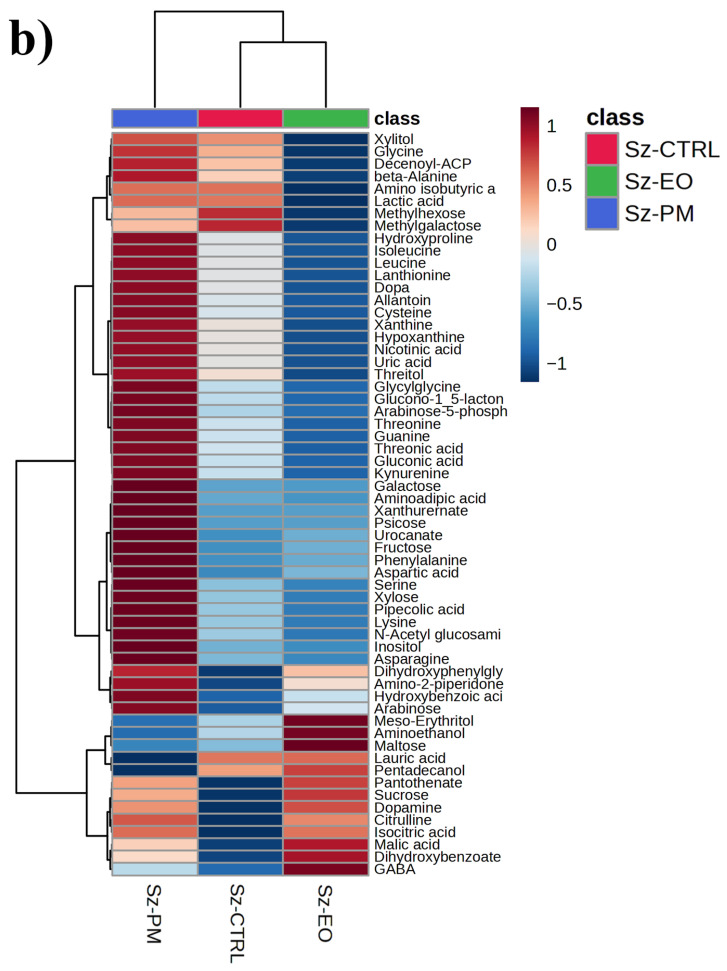
Top 60 metabolites resulting from the one-way ANOVA are reported as a clustered heatmap in (**a**) the adult stage of *Prostephanus truncatus* (Pt) and (**b**) *Sitophilus zeamais* (Sz) treated with *Commiphora myrrha* essential oil (EO), the insecticide pirimiphos-methyl (PM) and control (CTRL). The top hierarchical clusterization was built using the Euclidean distance and Ward as the clusterizing algorithm. The full list of significantly altered metabolites resulting from the ANOVA is reported in the excel file defined as Table S1. N = 5.

After PCA and PLS-DA, we applied a one-way ANOVA across all features to identify the top 60 metabolites most significantly altered by treatment and visualized their relative abundances in heatmaps (the full list of metabolites significantly affected is reported in Table S1). In *P. truncatus* (Figure 3a), samples segregate cleanly by treatment, controls cluster together, while *C. myrrha* EO- and PM-exposed insects form distinct branches, reflecting three metabolite modules: one comprising sugars, sugar alcohols, phosphorylated intermediates and certain amino acid derivatives that are low in controls but strongly up-regulated under EO (intense “hot” coloration in EO columns); a second of organic acids, nucleobase catabolites and a subset of amino acids elevated specifically under PM; and a central set of metabolites suppressed by both treatments relative to controls, indicating a shared stress response. These patterns, treatment-specific up-regulation of largely non-overlapping metabolite sets alongside a common down-regulated core, complement the multivariate findings by highlighting the distinct biochemical pathways mobilized by botanical versus synthetic insecticides. In *S. zeamais* (Figure 3b), the 60 ANOVA-selected metabolites are similarly partitioned into three categories: those that are markedly elevated only under EO, those that rise specifically with PM exposure, and a group that is consistently suppressed under EO.

**Figure 4:**
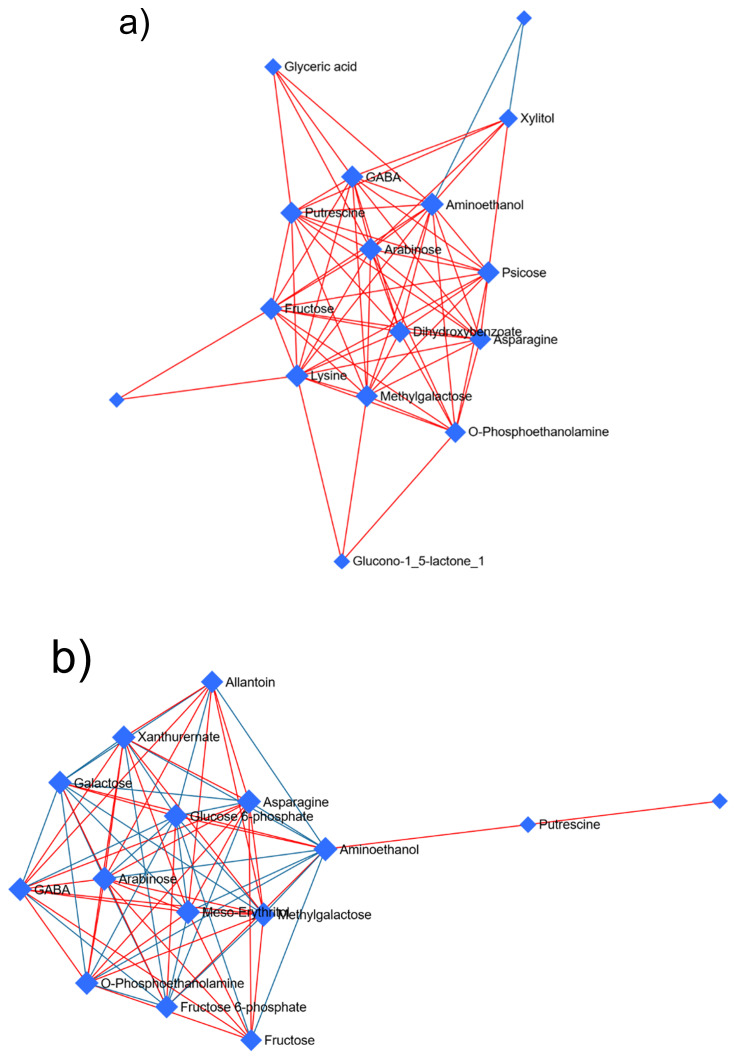

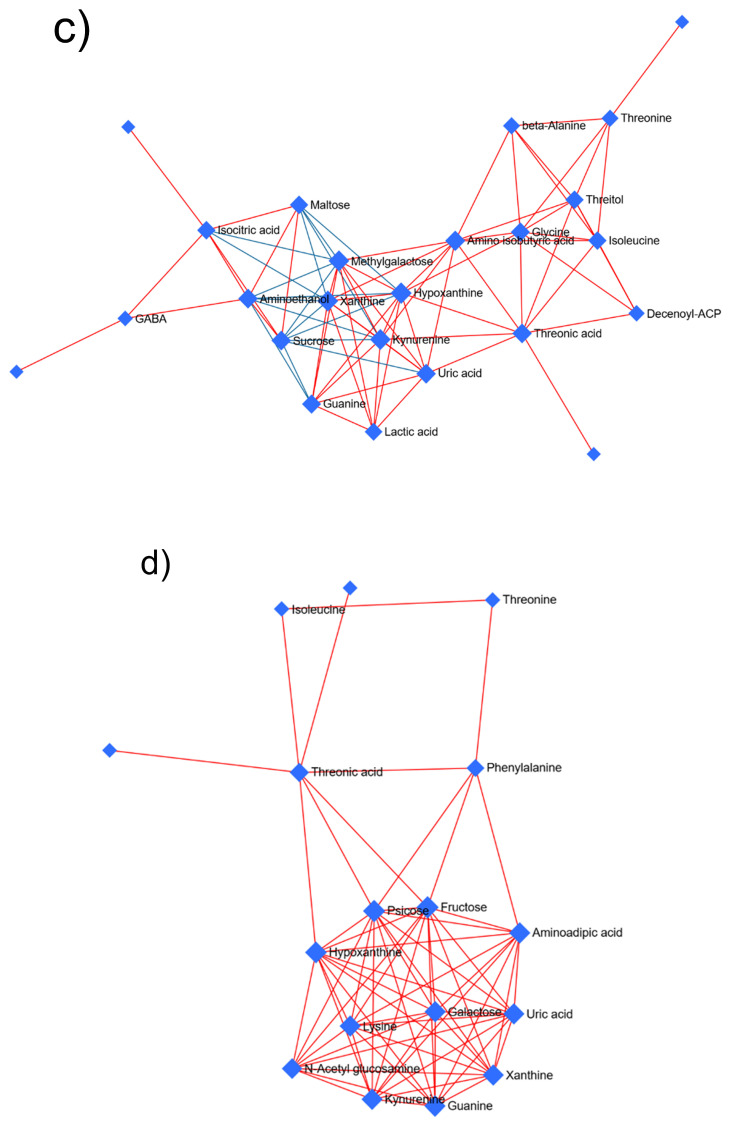
Correlation network obtained by the DSPC algorithm using the metabolites identified in the adult stage of *Prostephanus truncatus* treated with (**a**) *Commiphora myrrha* essential oil and (**b**) pirimiphos-methyl, and those identified in *Sitophilus zeamais* also treated with (**c**) *Commiphora myrrha* essential oil and (**d**) pirimiphos-methyl. Blue nodes represent metabolites, red lines indicate a positive correlation between metabolites, and blue lines indicate a negative correlation. N = 5.

Within *P. truncatus* exposed to *C. myrrha* EO (Figure 4a), a DSPC network of 16 metabolites converges on aminoethanol as the principal hub, its thirteen connections and betweenness centrality more than triple those of any other node, thereby linking sugar, amino acid and phosphorylated-intermediate clusters; lysine follows closely with twelve links and substantial betweenness, bridging a simple sugar community (fructose, arabinose, psicose, methylgalactose, xylitol) to nitrogenous amines (GABA, putrescine, asparagine), while highly connected carbohydrates (fructose, arabinose) underscore the centrality of carbohydrate flux and O-phosphoethanolamine anchors lipid-related pathways; peripheral nodes such as glyceric acid and glucono-1,5-lactone, with minimal degree and zero betweenness, occupy specialized downstream roles. Under the organophosphate (PM) treatment (Figure 4b), aminoethanol again sits at the center, directly linked to a core of sugars and phosphates (arabinose, glucose-6-phosphate, meso-erythritol, methylgalactose, O-phosphoethanolamine), with GABA, galactose and xanthurernate forming a secondary layer and scantly connected metabolites such as allantoin and fructose perching at the edges. Here, putrescine’s modest connectivity belies a high betweenness, marking it as a critical conduit for metabolic traffic. Thus, both *P. truncatus* networks revolve around the same sugar–amine hub, yet EO recruits additional amino acids and aromatic compounds, whereas PM remains more tightly focused on core carbohydrate routes. In *S. zeamais*, EO treatment again positions aminoethanol at the network’s nexus, uniting a dense core of sugars and nucleotide derivatives (sucrose, guanine, xanthine, hypoxanthine, kynurenine) and organic-acid by-products (lactate, urate) with an amino acid subnetwork (isoleucine, threonine, glycine, β-alanine, amino-isobutyric acid, threitol) and a peripheral fatty acid intermediate (decenoyl-ACP), while GABA, despite few direct links, exhibits high betweenness between sugar–nucleotide and amino acid modules (Figure 4c). Under PM, however, hypoxanthine becomes the central hub in *S. zeamais*, anchoring connections among sugars, sugar-related compounds and purine bases, with a small branch of amino acids (threonic acid, threonine, phenylalanine, isoleucine) and distal fatty acid by-products (Figure 4d). Overall, both species redirect metabolism around sugar–amine scaffolds under insecticidal stress; EO elicits broader, more diverse network rewiring, especially in *S. zeamais*, which shows greater sensitivity to EO, whereas PM effects remain more narrowly confined to central carbon and purine pathways.

## 3. Discussion

The chemical composition of *C. myrrha* EO is linear with those previously reported [19,29,37] where furanoeudesma-1,3-diene and lindestrene resulted the main constituents (34–41.40 and 12–14% of the total EO composition, respectively). As regards isofuranodiene, Marongiu et al. [37] and Morteza-Semnani and Saeedi [38] did not report this compound in the chemical composition. This could be linked to the high temperatures occurring during distillation of the oleo-gum-resin and consequent GC-MS analysis, which usually lead this compound to convert into curzerene [29,39]. Thus, to overcome this analytical issue, furanoeudesma-1,3-diene, isofuranodiene, and curzerene were quantified using HPLC-DAD analysis, which confirmed the presence of curzerene in the EO. Mortality bioassays revealed varying toxicity responses of *S. zeamais* and *P. truncatus* adults upon exposure to *C. myrrha* EO at two concentrations (500 ppm and 1000 ppm) over a seven-day period. At the end of the trials, *P. truncatus* experienced elevated mortality, while *S. zeamais* showed moderate mortality.

The mode of action of *C. myrrha* EO is still unknown. However, the lipophilicity of furanosesquiterpenes could favor their penetration in the insect body and enhance their effects [40]. The metabolomic analysis conducted on mosquito larvae by Spinozzi et al. [29] treated with *C. myrrha* EO and its main constituents revealed that many metabolic responses were activated. Specifically, these products seemed to affect amino acid metabolism, modifying the pathways related to aspartate, alanine, and glutamate [29]. Metabolomic analysis of *C. myrrha* EO-treated tenebrionids revealed pronounced metabolic perturbations, affecting pathways related to sugar metabolism, neurotransmission, oxidative stress, and energy homeostasis [28]. When the furanosesquiterpene isofuranodiene was tested, after isolation and purification, against *P. truncatus*, it resulted in 56.7% mortality, at 1000 ppm, seven days after exposure [41]. Here, the mortality rate of *P. truncatus* after a seven-day exposure to 1000 ppm of the *C. myrrha* EO, characterized by the same furanosesquiterpene, was 85.6%. Given that the exposure to one of the constituents of the tested EO resulted in 48.5% of the mortality caused by the EO, isofuranodiene impairs *P. truncatus* adults. The questions of why this is not a fact about *S. zeamais* adults, or whether the EO remains efficient against *P. truncatus* when both species coexist, require further investigation.

Regarding the metabolomic findings, our GC-MS-driven untargeted profiling revealed several notable patterns, highlighting both species-specific and treatment-dependent metabolic shifts. This observation aligns with previous reports demonstrating that distinct species maintain divergent metabolic signatures under similar rearing conditions [28,42]. The strong species effect underscores the necessity of evaluating botanical insecticides on multiple target pests, since intrinsic biochemical differences can dictate susceptibility and detoxification capacity [43]. Even when the *C. myrrha* EO was tested against two species of the same family (Tenebrionidae), different metabolic disruptions were observed [28].

PLS-DA of *P. truncatus* metabolomes reveals that *C. myrrha* EO and organophosphate treatments induce mechanistically distinct disturbances. Although some metabolites showed similar directional changes, their magnitude and network connectivity differed between treatments. In particular, EO chiefly perturbed primary carbon metabolism (notably, sugars and sugar phosphates), while PM more strongly affected nitrogen and purine metabolism. These results suggest that insecticide exposure forces a profound metabolic reprogramming that diverts resources from growth toward survival-promoting pathways. In particular, both EO and PM increased a high accumulation, stronger in PM than EO, of several metabolites such as xanthurenate, arabitol, meso-erythritol, o-phosphopantotenate, galactose, hypoxanthine, nicotinic acid, glycylglycine, sarcosine and xanthyne. These results suggest that the insecticide exposure forces a profound metabolic reprogramming in insects, one that channels resources into survival promoting pathways rather than normal growth. For instance, permethrin-treated *Drosophila* shunt tryptophan into the kynurenine pathway, leading to xanthurenate accumulation, a response linked to bolstering NAD^+^ pools and quenching oxidative stress [44]. At the same time, sugar alcohols such as arabitol and meso erythritol rise markedly, reflecting their dual roles as osmoprotectants and scavengers of reactive oxygen species [45]. The concurrent increase in O phosphopantotenate and nicotinic acid signals an upsurge in CoA and NAD^+^ biosynthesis, essential cofactors for phase II detoxification enzymes and redox reactions [44]. Disruptions in carbohydrate metabolism are evident too: elevated galactose and hypoxanthine levels in imidacloprid exposed aphids point to impaired glycolytic flux and accelerated ATP turnover under oxidative duress [46]. Finally, the rise of dipeptides such as glycylglycine and one carbon donor such as sarcosine highlights intensified proteolysis and methylation activity, mechanisms by which damaged proteins are recycled and cellular repair is fueled [44]. Together, these shifts illustrate a coordinated “all-hands-on-deck” strategy, whereby insects divert central carbon and amino acid metabolism to meet the energetic and antioxidant demands imposed by chemical assault. Similarly, in the case of *A. diaperinus*, the highly effective *C. myrrha* EO (91.1% larval mortality) extensively shifted the carbohydrate metabolism of larvae [28].

In any case, considering both the multivariate analysis and ANOVA results, it was highlighted that EO chiefly perturbs the primary carbon pathways (notably, sugars and sugar phosphates), whereas the organophosphate affects nitrogen and purine metabolism, underscoring non-overlapping metabolic targets for these two agents.

The extensive shifts in primary carbon and nitrogen metabolism observed in EO-treated *P. truncatus* underscore how disruption of these fundamental pathways can undermine insect physiology. Nitrogen, a core component of proteins, nucleic acids, and other macromolecules, is indispensable not only for cellular structure but also for energy production and immune, reproductive, and stress-response functions [47,48,49,50,51,52,53]. Likewise, central carbon metabolites, glucose 6 phosphate among them, mediate the allocation of resources under nutrient fluctuation, enabling organisms to maintain vital functions when challenged [54,55]. The prominence of both sugar phosphates and amino acid derivatives such as kynurenic acid among VIP > 1 metabolites thus reflects targeted disturbances to carbon and nitrogen flux under EO stress.

Network-level analysis further clarifies these effects: in the EO-treated DSPC network, aminoethanol, lysine, and key sugars form a dense hub, implicating ethanolamine-linked pathways, including phospholipid headgroup turnover and membrane remodeling, as critical vulnerabilities to furanosesquiterpene toxicity [56]. Under PM treatment, although aminoethanol remains central, the elevated betweenness of putrescine suggests a rerouting of metabolic flux toward polyamine catabolism and reactive oxygen species detoxification [57,58,59]. In summary, both insecticides converge on an aminoethanol, sugar scaffold; however, myrrh EO provokes broader remodeling, extending into aromatic amino acid (tyrosine, phenylalanine) and lysine pathways, whereas PM exerts a more focused effect on carbohydrate–phosphate and polyamine hubs.

In *S. zeamais*, EO exposure provoked a broader and more complex metabolic remodeling compared to *P. truncatus*. The strongest alterations were observed in carbohydrate metabolism, amino acid pathways, and nucleotide turnover, indicating a multifaceted disruption of both energy and nitrogen fluxes. By contrast, PM treatment produced a narrower metabolic profile, largely converging on purine degradation and oxidative stress markers. These patterns highlight that EO induces a flexible and diversified reprogramming, while PM exerts a more constrained but energetically demanding effect.

In fact, in *S. zeamais*, *C. myrrha* essential oil (EO) triggers an extensive metabolic overhaul that surpasses even that seen in *P. truncatus*. PLS-DA of *S. zeamais* reveals a larger LV1 shift with 53 VIP > 1 metabolites dominated by sugars (sucrose, fructose, arabinose), phosphorylated compounds (glycerol α phosphate), amino acids (aspartate, citrulline, histidine, phenylalanine, homocysteine) and nucleotide catabolites (urocanate), indicating that EO delivers a multifaceted assault on both energy metabolism and nitrogen flux, as also observed by Spinozzi et al. [29] in mosquitoes. Concomitant rises in TCA intermediates (malic and isocitric acids), and cofactor pools (pantothenate) indicate potentially intensified ATP production, NADH regeneration, and coenzyme A synthesis to support broad Phase II detoxification and membrane repair [60,61]. Neuro metabolites such as dopamine, tryptamine and hydroxy DL kynurenine further reflect the activation of shikimate and kynurenine routes, underscoring antioxidant and signaling responses under EO stress [62].

By contrast, PM elicits a sharper, more constrained disruption. Although sugars and TCA intermediates become extremely elevated, an energy crisis signature of irreversible acetylcholinesterase inhibition, PLS DA and targeted profiling highlight a predominant convergence on purine degradation pathways, suggesting focused perturbation of nucleotide homeostasis under organophosphate pressure [63,64,65]. Intermediate increases in phosphorylated metabolites, amino acids and biogenic amines indicate that PM provokes some broader metabolic strain, but without the flexible reprogramming seen under EO. The one-way ANOVA heatmaps reinforce these treatment- and species-specific patterns. In *P. truncatus*, EO uniquely elevated a cluster of sugars and sugar alcohols, such as xylitol and sorbitol, suggesting increased pentose phosphate pathway activity and osmoprotectant synthesis under myrrh EO exposure [66]. PM, by contrast, raised levels of organic acids (aconitic and decanoic acids) and purine catabolites, consistent with mitochondrial dysfunction and oxidative stress [44,67]. Likewise, in *S. zeamais*, EO drove the up-regulation of aromatic amino acids (tyrosine, tryptophan) and kynurenine pathway intermediates, pointing to possible neural or neurotransmitter disruption, whereas PM treatment principally increased xanthine and uric acid, hallmarks of oxidative damage in nucleic acid turnover [44,68,69,70].

## 4. Materials and Methods

### 4.1. Chemical Analysis of C. myrrha Essential Oil (EO)

The *C. myrrha* EO was purchased from El Taller De Alquimia, S.L. (Tortella, Girona, Spain) and was qualitatively analyzed with an Agilent 8890 gas chromatograph coupled to a 5977B single quadrupole mass spectrometer (Santa Clara, CA, USA) and an autosampler PAL RTC120 (CTC Analytics AG, Zwingen, Switzerland). The EO was diluted 1:100 in diethyl ether and 1 µL injected in split mode (1:200). The ionization was achieved using an electron ionization source (EI), while the injector temperature was 280 °C. Helium was used as the gas carrier at a flow rate of 1 mL min-1. For the chromatographic separation, an HP-5MS capillary column (30 m, 250 µm i.d., 0.25 µm film thickness) was used, being thermostated at 60 °C for 5 min and then raised up to 220 °C at 4 °C/min, then to 280 °C at 11 °C/min, and held for 15 min, and finally to 300 °C at 15 °C/min and held for 0.5 min. The run time was about 67 min. The transfer line temperature was 280 °C and the temperature of the ionization source and the mass analyzer were set at 230 and 150 °C, respectively. The acquisition was carried out in SCAN mode (29–400 *m*/*z*). Chromatograms were analyzed using the MSD ChemStation software (Agilent, Santa Clara, California, U.S.A., Version G1701DA D.01.00) and the NIST Mass Spectral Search Program for the NIST/EPA/NIH EI and NIST Tandem Mass Spectral Library v. 2.3 were used for data analysis. The identification of sample components was achieved through the comparison of the temperature-programmed retention indices (Ris) and mass spectra (MS) of each peak with those in bibliography [71,72,73].

Regarding the quantification of the main furanosesquiterpenoids of the EO (furanoeudesma-1,3-diene, curzerene and isofuranodiene), it was performed using an Agilent Technologies high performance-liquid chromatograph (HPLC) HP-1100 series (Palo Alto, CA, USA), constituted by an autosampler, a binary solvent pump, and a diode array detector (DAD). The molecules were separated through a Kinetex^©^ PFP 100A column (100 × 4.6 mm internal diameter, 2.6 μm) purchased from Phenomenex (Torrance, CA, USA). The analytical conditions as well as the quantification of the analytes were performed as reported by Spinozzi et al. [29].

### 4.2. Insects Rearing and Grain Substrate

In the Laboratory of Agricultural Zoology and Entomology at the Agricultural University of Athens, stored-product insect colonies were maintained under complete darkness. *Prostephanus truncatus* and *S. zeamais* were cultured using whole maize. All colonies were maintained at a constant temperature of 30 °C with 65% relative humidity (RH). No sex determination was conducted for the selected individuals. Adults of *S. zeamais* and *P. truncatus* were selected within 14 [74] and 7 [75] days of age, respectively. For the mortality bioassays, pesticide-free maize, *Zea mays* L. (var. Dias), was utilized as the substrate. Prior to experimentation, the moisture content of maize was measured at 12.9% using the C-Pro grain moisture meter (AgroLog, Søndersø, Denmark).

### 4.3. Mortality Bioassays

After conducting preliminary tests, concentrations of 500 and 1000 ppm (500 and 1000 μL EO/kg maize, respectively) were selected for the trials. Solutions contained 150 μL and 250 μL of EO, which were first combined with pure ethanol in a 1:1 ratio (*v*/*v*) to achieve the respective concentrations. Tween 80 was then introduced as a 0.3% (*v*/*v*) aqueous solution, bringing the total volume to 750 μL (Table 4). The solutions were applied using a GHPM-Mobius airbrush (Gaahleri, NJ, USA), evenly spraying 0.25 kg of maize per treatment on separate trays. Furthermore, maize lots of 0.25 kg were sprayed with negative controls, i.e., distilled water, pure ethanol, pure ethanol in water and Tween 80 (carrier control), or a positive control, i.e., Actellic EC containing 50% PM at the recommended concentration of 5 ppm (5 μL/kg maize) (Table 4). To prevent contamination, a separate airbrush was designated for each scenario. Once treated, the maize samples were placed in 1 L glass jars and were manually agitated for 10 min to ensure the uniform distribution of the applied solutions [41]. From every jar, three subsamples of 10 g (sub-replicates) were taken using a clean spoon for each treatment. These portions were precisely weighed using a Precisa XB3200D electronic balance (Alpha Analytical Instruments, Gerakas, Greece). The sub-replicates were then placed in glass vials (125 mm height × 75 mm diameter) embedded with ventilated caps (opening sealed with fabric) to allow airflow. To prevent insect escape, the inner walls near the caps were coated with a polytetrafluoroethylene dispersion in water (60% by weight) (Sigma-Aldrich Chemie GmbH, Schnelldorf, Germany). In each glass vial, 10 adult insects were assigned, corresponding to the negative controls, positive control (PM), and EO treatments (500 and 1000 ppm). Afterward, the vials were incubated under the predetermined conditions (as specified in the subsection titled Insects rearing and grain substrate). Mortality assessments were conducted at intervals of 8–24 h and then from 2 to 7 days utilizing an Olympus SZX9 stereomicroscope (Bacacos S.A., Athens, Greece). A fine brush (different per treatment) was used to detect movement in insect individuals. To maintain experimental integrity, the entire procedure was repeated twice more with fresh sets of glass vials, insects, and maize for each replication [76].

### 4.4. Metabolomic Analysis Bioassays

For each treatment, approximately 0.3 g of insects (individuals of species/life stage) were introduced into glass jars containing 0.25 kg of maize that had been sprayed with control (distilled water—negative control), 5 ppm PM (positive control), or 1000 ppm of *C. myrrha* EO. The jars were then transferred to incubators set to the predetermined abiotic conditions (subsection: Insects rearing and grain substrate). Insects from each treatment and replication were carefully collected from the treated maize and placed into individually labeled plastic tubes (0.1 × 0.016 cm) after 1 day of exposure. These tubes were immediately immersed in liquid nitrogen to ensure rapid preservation. Right after freezing, they were transferred to a −80 °C freezer, where they remained until the initiation of metabolomic analysis. To ensure reliability, the entire experiment was repeated four additional times, each time using fresh sets of glass jars, insects, and maize.

### 4.5. Metabolite Extraction and GC–MS Untargeted Metabolomic Analysis

To extract and profile metabolites from both larval and adult stages, the protocol of Spinozzi et al. [29] was adapted, introducing few custom tweaks. Tissue samples (100 mg) were flash-frozen in liquid nitrogen and pulverized into a fine powder prior to extraction. Each sample was treated with 1400 μL of pre-chilled methanol (−20 °C) and supplemented with 60 μL of ribitol solution (0.2 mg/mL in water), which served as the internal standard for the polar fraction. After vortexing for 10 s, the mixtures were incubated in a thermomixer at 70 °C with agitation at 950 rpm for 10 min, followed by centrifugation at 11,000× *g* for 10 min. The supernatant was carefully transferred to glass vials, where 750 μL of cold chloroform (−20 °C) and 1500 μL of chilled water (4 °C) were sequentially added. After another 10 s vortex step, the samples were centrifuged at 2200× *g* for 15 min.

From each extract, 150 μL of the polar layer was recovered into 1.5 mL tubes and dried in a vacuum concentrator without heat. For derivatization, 40 μL of methoxyamine hydrochloride (20 mg/mL in pyridine; Merck Life Science, Milan, Italy) was added, and the samples were incubated at 37 °C with shaking at 950 rpm for 2 h. Silylation was then performed by adding 70 μL of MSTFA (Merck Life Science, Milan, Italy) and shaking at 37 °C for 30 min. Finally, 110 μL of each derivatized extract was transferred into GC/MS-compatible glass vials for subsequent analysis.

After derivatization, samples were run on an Agilent (Santa Clara, CA, USA) 8890 gas chromatograph linked to a 5977C single quadrupole mass spectrometer, with injections handled by a CTC PAL autosampler. Chromatographic separation took place on a 30 m × 0.25 mm × 0.25 µm 5MS capillary column, preceded by a 10 m guard column.

All oven temperature ramps, carrier-gas flow rates, inlet settings and MS acquisition parameters followed the detailed scheme of Misra [77]. Specifically, the injector temperature was maintained at 250 °C, while the ion source was set to 260 °C. For each run, a 1 µL portion of the sample was introduced in splitless mode, using helium as the carrier gas at a constant flow rate of 1 mL/min. The oven program began with an isothermal step at 70 °C for 5 min, followed by a ramp of 5 °C per minute up to 350 °C, and concluded with a 5 min hold at 330 °C. Mass spectral data were collected under electron ionization (EI) conditions at 70 eV, scanning the range of 40–600 *m*/*z* with a scan interval of 0.2 s. A solvent delay of 9 min was applied. To ensure reliable performance and accurate compound identification, samples, n-alkane reference standards, and pyridine blanks were injected at intervals to check instrument stability and detect any shifts in retention indices (RI). Between analytical runs, solvent blanks were introduced to assess possible contamination, and all detected masses were cross-checked against these blanks to exclude background interferences.

Data processing, including baseline correction, feature alignment, spectral deconvolution, peak picking and putative metabolite annotation, was performed in MS-DIAL. To ensure consistency, we periodically ran solvent blanks, pooled quality-control samples, and a series of odd-chain n-alkanes (C_10_ to C_40_) to track retention-index shifts and instrument drift. Metabolite IDs were assigned by matching experimental spectra and retention indices against our in-house electron ionization library, and we reported compounds at confidence levels 2 or 3 in line with Sumner et al. [78].

### 4.6. Data Analysis

Since mortality in the negative control group remained <5% for all tested individuals, no corrections were required. Before statistical analysis, the dataset underwent log (x + 1) transformation to normalize variance [79,80]. Each species was analyzed separately using the repeated measures model [81], accounting for related interactions of the main effects examined. The main effects were treatment and concentration, mortality was the response variable, and exposure time was the repeated factor. Statistical analyses were performed using JMP 16.2 [82]. Mean separation was conducted at a significance level of 0.05 using a Tukey test (HSD) [83]. Additionally, a two-tailed *t*-test [84] was applied at n—2 df and the 0.05 level to compare each EO concentration with the positive control of each treatment for each species.

Metabolomic analyses were carried out under a completely randomized layout with five biological replicates per treatment. Raw intensity values from MS-DIAL were first normalized to the ribitol internal standard, then Log_10_-transformed and Pareto-scaled using MetaboAnalyst 6.0. To explore the overall data structure without prior group assignment, we applied unsupervised principal component analysis (PCA). We then used supervised partial least-squares discriminant analysis (PLS-DA) to focus on metabolites that best separate our control, EO and PM groups. In both PCA and PLS-DA score plots, the Hotelling’s T^2^ ellipse denotes the 95% confidence region. In PLS-DA analysis, metabolites with a VIP (variable importance in projection) score greater than 1 were flagged as key discriminators. To guard against overfitting, we performed a 20-permutation test, accepting the model only if permuted Q^2^ and R^2^Y values remained below 0.05 and trended toward unity. Next, we calculated pairwise partial correlations among metabolites using the debiased sparse partial correlation (DSPC) method (betweenness filter cutoff = 1), which uncovers direct relationships between metabolite pairs while accounting for indirect links. We also conducted one-way ANOVA on each feature (*p* ≤ 0.05), correcting for multiple comparisons via the False Discovery Rate (FDR ≤ 0.05), to identify those metabolites whose levels change significantly with treatment. The top 60 metabolites resulting from the ANOVA analysis are graphically reported in the text as a heatmap, whereas the full list of significantly altered metabolites is reported in the Supplementary Table S1.

## 5. Conclusions

This study provided new insights for the application of myrrh EO as a natural protectant of stored maize. Its main furanosequiterpenes, such as furanoeudesma-1,3-diene, curzerene, lindestrene, and isofuranodiene, were revealed to be ideal candidate ingredients for use in formulations on maize due to their inhibitory properties against the two stored-product pests. The availability on the market of myrrh EO to satisfy the needs of cosmetic, pharmaceutical, and nutraceutical applications may pave the way for an additional supply of myrrh EO for the agrochemical industry. The metabolomic alterations documented here elucidate how *C. myrrha* EO triggers species- and treatment-specific metabolic cascades in two major stored-product pests. The contrasting profiles between EO and PM treatments further underscore the value of metabolomics for unravelling complex modes of action. By spotlighting key metabolic hubs—aminoethanol, sugar phosphates, aromatic amino acids, and purine catabolites—we provide a foundational framework for future efforts to optimize botanical insecticides, design synergists that block compensatory pathways, and develop diagnostic markers of insecticide exposure and resistance emergence.

## Figures and Tables

**Table 1:** MANOVA parameters showing the main effects and their interactions leading to the observed mortalities of *Sitophilus zeamais* and *Prostephanus truncatus* adults (error df = 32 for all species).

		Between Exposures				Within Exposures			
		Intercept	Concentration	Formulation	Concentration x Formulation	Exposure	Exposure x Concentration	Exposure x Formulation	Exposure x Concentration x Formulation
df		1	1	1	1	9	9	9	9
*S. zeamais* adults	*F*	2957.5	27.2	1378.0	23.9	3616.5	131.1	2027.7	135.2
	*p*	<0.01	<0.01	<0.01	<0.01	<0.01	<0.01	<0.01	<0.01
*P. truncatus* adults	*F*	3369.5	7.9	18.6	4.8	7263.7	7.6	39.3	8.0
	*p*	<0.01	0.01	0.01	0.04	<0.01	<0.01	<0.01	<0.01

**Table 2:** Mean (%) mortality ± standard errors (SE) of *Sitophilus zeamais* adults after 4–16 hours (h), and 1–7 days (d) in maize treated with *Commiphora myrrha* essential oil (EO) or pirimiphos-methyl (PM) (positive control).

	*C. myrrha* EO	PM			*C. myrrha* EO	PM		
Dose	500 ppm				1000 ppm			
Exposure			*t*	*p*			*t*	*p*
4 h	0.0 ± 0.0 B	0.0 ± 0.0 E	-	-	0.0 ± 0.0 C	0.0 ± 0.0 E	-	-
8 h	0.0 ± 0.0 B	0.0 ± 0.0 E	-	-	0.0 ± 0.0 C	0.0 ± 0.0 E	-	-
16 h	0.0 ± 0.0 B	4.4 ± 2.4 D	2.0	0.08	0.0 ± 0.0 C	5.6 ± 2.4 D*	2.5	0.02
1 d	0.0 ± 0.0 B	13.3 ± 2.9 C*	7.3	<0.01	0.0 ± 0.0 C	13.3 ± 3.3 C*	5.1	0.01
2 d	0.0 ± 0.0 B	30.0 ± 4.4 B*	27.2	<0.01	0.0 ± 0.0 C	31.1 ± 4.2 B*	29.1	<0.01
3 d	0.0 ± 0.0 B	46.7 ± 3.3 AB*	61.3	<0.01	0.0 ± 0.0 C	48.9 ± 3.1 AB*	67.4	<0.01
4 d	0.0 ± 0.0 B	58.9 ± 3.1 AB*	84.2	<0.01	0.0 ± 0.0 C	61.1 ± 3.1 AB*	85.6	<0.01
5 d	0.0 ± 0.0 B	77.8 ± 2.8 A*	125.5	<0.01	10.0 ± 1.7 B	78.9 ± 2.6 A*	7.6	<0.01
6 d	0.0 ± 0.0 B	92.2 ± 2.2 A*	187.9	<0.01	18.9 ± 1.1 A	91.1 ± 2.0 A*	20.6	<0.01
7 d	10.0 ± 1.7 A	100.0 ± 0.0 A*	8.5	<0.01	25.6 ± 2.4 A	100.0 ± 0.0 A*	16.0	<0.01
*F*	60.0	107.8			203.9	82.7		
*p*	<0.01	<0.01			<0.01	<0.01		

Values are means (±standard errors). For each exposure, within each row, asterisks indicate significant differences (df = 16; two-tailed *t*-test at *p* = 0.05). For each concentration, within each column, means followed by the same uppercase letter are not significantly different (df = 9, 89; Tukey HSD test at *p* = 0.05). No significant differences were recorded where no asterisks or letters exist. No statistical analysis was performed where dashes exist.

**Table 3:** Mean (%) mortality ± standard errors (SE) of *Prostephanus truncatus* adults after 4–16 h and 1–7 days (d) in maize treated with *Commiphora myrrha* essential oil (EO) or pirimiphos-methyl (PM) (positive control).

	*C. myrrha* EO	PM			*C. myrrha* EO	PM		
Dose	500 ppm				1000 ppm			
Exposure			*t*	*p*			*t*	*p*
4 h	0.0 ± 0.0 E	0.0 ± 0.0 E	-	-	0.0 ± 0.0 G	0.0 ± 0.0 E	-	-
8 h	0.0 ± 0.0 E	2.2 ± 1.5 DE	1.5	0.17	0.0 ± 0.0 G	3.3 ± 1.7 DE	2.0	0.06
16 h	4.4 ± 1.8 D	4.4 ± 1.8 D	0.0	1.00	7.8 ± 1.5 F	5.6 ± 1.8 D	−1.0	0.35
1 d	10.0 ± 1.7 C	16.7 ± 2.4 C	1.0	0.34	14.4 ± 1.8 E	16.7 ± 2.9 C	−0.2	0.83
2 d	15.6 ± 2.9 BC	33.3 ± 2.9 BC*	2.8	0.02	25.6 ± 1.8 D	34.4 ± 3.4 BC*	2.3	0.03
3 d	20.0 ± 1.7 ABC	50.0 ± 1.7 AB*	9.6	<0.01	32.2 ± 1.5 CD	51.1 ± 1.1 AB*	10.0	<0.01
4 d	26.7 ± 2.4 AB	61.1 ± 2.0 AB*	9.3	<0.01	42.2 ± 2.2 BCD	62.2 ± 1.5 AB*	6.7	<0.01
5 d	30.0 ± 1.7 AB	77.8 ± 2.8 AB*	14.2	<0.01	55.6 ± 2.4 ABC	78.9 ± 3.9 AB*	5.5	<0.01
6 d	38.9 ± 3.1 A	88.9 ± 3.1 A*	8.6	<0.01	70.0 ± 1.7 AB	90.0 ± 2.9 AB*	6.2	<0.01
7 d	48.9 ± 3.5 A	97.8 ± 1.5 A*	10.3	<0.01	85.6 ± 2.4 A	96.7 ± 1.7 A*	3.8	0.01
*F*	53.0	72.3			182.7	61.4		
*p*	<0.01	<0.01			<0.01	<0.01		

Values are means (±standard errors). For each exposure, within each row, asterisks indicate significant differences (df = 16; two-tailed *t*-test at *p* = 0.05). For each concentration, within each column, means followed by the same uppercase letter are not significantly different (df = 9, 89; Tukey HSD test at *p* = 0.05). No significant differences were recorded where no asterisks or letters exist. No statistical analysis was performed where dashes exist.

**Table 4:** Solutions of the four treatments conducted against *P. truncatus* and *S. zeamais* adults.

Treatment	Solutions
*C. myrrha* EO 500 ppm	150 μL EO + 150 μL ethanol + 450 μL Tween 80 0.3% (*v*/*v*)
*C. myrrha* EO 1000 ppm	250 μL EO + 250 μL ethanol + 250 μL Tween 80 0.3% (*v*/*v*)
Positive control	5 ppm pirimiphos-methyl
Carrier control	150 μL distilled water + 150 μL pure ethanol + 150 μL pure ethanol in water + 300 μL Tween 80 0.3% (*v*/*v*)

## Data Availability

Data are available within the article.

## Supplementary Materials

The following supporting information can be downloaded at: https://www.mdpi.com/article/10.3390/plants14193031/s1, Table S1: Statistical metabolomics dataset providing comprehensive details on the raw, untargeted GC-MS measurements along with all associated statistical parameters.

## References

[1] Langenheim J.H. (2003). Plant Resins: Chemistry, Evolution, Ecology, and Ethnobotany.

[2] Mutiga I.M. (2021). Ecological restoration of pastoral landscapes in the drylands of East Africa. J. Dryland Agric..

[3] Başer K.H.C., Demirci B.E.T.Ü.L., Dekebo A., Dagne E. (2003). Essential oils of some *Boswellia* spp., myrrh and opopanax. Flavour Fragr. J..

[4] Alsherif E.A. (2019). Ecological studies of *Commiphora* genus (myrrha) in Makkah region, Saudi Arabia. Heliyon.

[5] Hanuš L.O., Řezanka T., Dembitsky V.M., Moussaieff A. (2005). Myrrh—*Commiphora* chemistry. Biomed. Papers.

[6] Shalabi L.F., Otaif F.S. (2022). *Commiphora* Jacq (Burseraceae) in Saudi Arabia, botanical, phytochemical and ethnobotanical notes. Ecologies.

[7] Hassan B., Glover E.K., Luukkanen O., Chikamai B., Jamnadass R., Iiyam M., Kanninen M. (2011). The role of *Boswellia* and *Commiphora* species in rural livelihood security and climate change adaptation in the Horn of Africa: Case study of northeastern Kenya. Int. J. Soc. For..

[8] Latha S., Selvamani P., Prabha T. (2021). Pharmacological uses of the plants belonging to the genus *Commiphora*. Cardiovasc. Hematol. Agents Med. Chem..

[9] Cao B., Wei X.C., Xu X.R., Zhang H.Z., Luo C.H., Feng B., Xu R.C., Zhao S.Y., Du X.J., Han L. (2019). Seeing the unseen of the combination of two natural resins, frankincense and myrrh: Changes in chemical constituents and pharmacological activities. Molecules.

[10] Abdul-Ghani R.A., Loutfy N., Hassan A. (2009). Myrrh and trematodoses in Egypt: An overview of safety, efficacy and effectiveness profiles. Parasitol. Int..

[11] Nohr L.A., Rasmussen L.B., Straand J. (2009). Resin from the mukul myrrh tree, guggul, can it be used for treating hypercholesterolemia? A randomized, controlled study. Complement. Ther. Med..

[12] Haffor A.S.A. (2010). Effect of myrrh (*Commiphora molmol*) on leukocyte levels before and during healing from gastric ulcer or skin injury. J. Immunotoxicol..

[13] Su S., Wang T., Duan J.A., Zhou W., Hua Y.Q., Tang Y.P., Yu L., Qian D.W. (2011). Anti-inflammatory and analgesic activity of different extracts of *Commiphora myrrha*. J. Ethnopharmacol..

[14] De Rapper S., Van Vuuren S.F., Kamatou G.P.P., Viljoen A.M., Dagne E. (2012). The additive and synergistic antimicrobial effects of select frankincense and myrrh oils—A combination from the pharaonic pharmacopoeia. Lett. Appl. Microbiol..

[15] Mehta A.K., Tripathi C.D. (2015). *Commiphora mukul* attenuates peripheral neuropathic pain induced by chronic constriction injury of sciatic nerve in rats. Nutr. Neurosci..

[16] Rahmani A.H., Anwar S., Raut R., Almatroudi A., Babiker A.Y., Khan A.A., Alsahli M., Almatroodi S.A. (2022). Therapeutic potential of myrrh, a natural resin, in health management through modulation of oxidative stress, inflammation, and advanced glycation end products formation using in vitro and in silico analysis. Appl. Sci..

[17] Goda A.A., Aya E.G., Amin M.H., Youssef M., Shi J., Xu J., Liu X., Zhou Y., Xiao L., Ramzy S. (2024). Evaluation of the antimicrobial and anticancer properties of myrrh resin extract and its application in cacao beverages. Discov. Food.

[18] Hamed A.M., Awad A.A., Abdel-Mobdy A.E., Alzahrani A., Salamatullah A.M. (2021). Buffalo yogurt fortified with eucalyptus (*Eucalyptus camaldulensis*) and myrrh (*Commiphora myrrha*) essential oils: New insights into the functional properties and extended shelf life. Molecules.

[19] Dekebo A., Juniedi S., Hu X., Jung C., Murthy H.N. (2021). Ethnobotany, chemistry, and biological activities of some *Commiphora* species resins. Gums, Resins and Latexes of Plant Origin: Chemistry, Biological Activities and Uses.

[20] Baz M.M., Khater H.F., Baeshen R.S., Selim A., Shaheen E.S., El-Sayed Y.A., Salama A.S., Hegazy M.M. (2022). Novel pesticidal efficacy of *Araucaria heterophylla* and *Commiphora molmol* extracts against camel and cattle blood-sucking ectoparasites. Plants.

[21] Dinku W., Park S.B., Jeong J.B., Jung C., Dekebo A. (2022). Chemical composition and anti-inflammatory activity of essential oils from resin of *Commiphora* species. Bull. Chem. Soc. Ethiop..

[22] Shah M., Khan F., Ullah S., Khan A., Alsabahi J.N., Zainab R., Bilal M., Khan S., Rafiq N., Rehman N.U. (2023). Chemical composition and biomedical effects of essential oil from *Commiphora foliacea* Sprague stem. J. Essent. Oil-Bear. Plants.

[23] Alanazi N.A.H., Alamri A.A., Mashlawi A.M., Almuzaini N., Mohamed G., Salama S.A. (2024). Gas chromatography–mass spectrometry chemical profiling of *Commiphora myrrha* resin extracts and evaluation of larvicidal, antioxidant, and cytotoxic activities. Molecules.

[24] Hosseinkhani A., Ghavidel F., Mohagheghzadeh A., Zarshenas M.M. (2017). Analysis of six populations of *Commiphora myrrha* (Nees) Engl. oleo-gum resin. Trends Pharm. Sci. Technol..

[25] Batiha G.E.S., Wasef L., Teibo J.O., Shaheen H.M., Zakariya A.M., Akinfe O.A., Teibo T.K.A., Alkuraishy H.M., Al-Garbee A.I., Alexiou A. (2023). *Commiphora myrrh*: A phytochemical and pharmacological update. Naunyn-Schmiedeberg’s Arch. Pharmacol..

[26] Al-Fuhaid N. (2017). Repellency and fumigant toxicity of *Aloe vera*, *Astragalus sarcocolla*, *Commiphora myrrha* and *Ferula assafoetida* L gum resin powders and methanol extracts against *Trogoderma granarium* Everts. Int. J. Agric. Sci..

[27] Wahba T.F., Aly H.M., Hassan N.A. (2023). The antifeedant properties of bio-oil from *Cupressus sempervirens* against rice weevil (*Sitophilus oryzae*) compared to that of myrrh and frankincense oils. Egypt. J. Agric. Res..

[28] Kavallieratos N.G., Boukouvala M.C., Gidari D.L.S., Filintas C.S., Skourti A., Maggi F., Ferrati M., Petrelli R., Spinozzi E., Teruzzi C. (2025). Advances in stored-product pests control: Evaluation of the efficacy of myrrh essential oil on two Tenebrionidae species through a metabolomic approach. J. Stord Prod. Res..

[29] Spinozzi E., Ferrati M., Baldassarri C., Rossi P., Favia G., Cameli G., Benelli G., Canale A., De Fazi L., Pavela R. (2025). Essential oil and furanosesquiterpenes from myrrh oleo-gum resin: A breakthrough in mosquito vector management. Nat. Prod. Bioprospect..

[30] Hagstrum D.W., Phillips T.W. (2017). Evolution of stored-product entomology: Protecting the world food supply. Annu. Rev. Entomol..

[31] Belluco S., Bertola M., Montarsi F., Di Martino G., Granato A., Stella R., Martinello M., Bordin F., Mutinelli F. (2023). Insects and public health: An overview. Insects.

[32] Rosentrater K.A. (2022). Storage of Cereal Grains and their Products.

[33] Muatinte B.L., Kavallieratos N.G., Boukouvala M.C., García-Lara S., López-Castillo L.M., Mvumi B.M., Hemming D. (2025). The threat of the larger grain borer, *Prostephanus truncatus* (Coleoptera: Bostrichidae) and practical control options for the pest. Invasive Species Reviews 2018–2024.

[34] Hacham Y., Hershenhorn J., Dor E., Amir R. (2016). Primary metabolic profiling of Egyptian broomrape (*Phelipanche aegyptiaca*) compared to its host tomato roots. J. Plant. Physiol..

[35] Cui S.F., Wang L., Qiu J.P., Geng X.Q., Liu Z.C. (2019). Effects of hypoxia/hypercapnia on the metabolism of *Callosobruchus chinensis* (L.) larvae. J. Stored Prod. Res..

[36] Ferri I., Dell’Anno M., Spano M., Canala B., Petrali B., Dametti M., Magnaghi S., Rossi L. (2024). Characterisation of *Tenebrio molitor* reared on substrates supplemented with chestnut shell. Insects.

[37] Marongiu B., Piras A., Porcedda S., Scorciapino A. (2005). Chemical composition of the essential oil and supercritical CO_2_ extract of *Commiphora myrrha* (Nees) Engl. and of *Acorus calamus* L.. J. Agric. Food Chem..

[38] Morteza-Semnani K., Saeedi M. (2003). Constituents of the essential oil of *Commiphora myrrha* (Nees) Engl. var. molmol. J. Essent Oil Res..

[39] Maggi F., Barboni L., Papa F., Caprioli G., Ricciutelli M., Sagratini G., Vittori S. (2012). A forgotten vegetable (*Smyrnium olusatrum* L., Apiaceae) as a rich source of isofuranodiene. Food Chem..

[40] Benelli G., Pavela R., Iannarelli R., Petrelli R., Cappellacci L., Cianfaglione K., Afshar F.H., Nicoletti M., Canale A., Maggi F. (2017). Synergized mixtures of Apiaceae essential oils and related plant-borne compounds: Larvicidal effectiveness on the filariasis vector *Culex quinquefasciatus* Say. Ind. Crops Prod..

[41] Kavallieratos N.G., Boukouvala M.C., Ntalli N., Skourti A., Karagianni E.S., Nika E.P., Kontodimas D.C., Cappellacci L., Petrelli R., Cianfaglione K. (2020). Effectiveness of eight essential oils against two key stored-product beetles, *Prostephanus truncatus* (Horn) and *Trogoderma granarium* Everts. Food Chem. Toxicol..

[42] Harrison B.R., Hoffman J.M., Samuelson A., Raftery D., Promislow D.E. (2022). Modular evolution of the *Drosophila* metabolome. Mol. Biol. Evol..

[43] Koul O., Singh R., Kaur B., Kanda D. (2013). Comparative study on the behavioral response and acute toxicity of some essential oil compounds and their binary mixtures to larvae of *Helicoverpa armigera, Spodoptera litura* and *Chilo partellus*. Ind. Crop Prod..

[44] Brinzer R.A. (2015). Drosophila, Metabolomics and Insecticide Action. Doctoral Dissertation.

[45] Storey K.B. (1997). Organic solutes in freezing tolerance. Comp. Biochem. Physiol. A Mol. Integr. Physiol..

[46] Lv N., Ma K., Li R., Liang P., Liang P., Gao X. (2021). Sublethal and lethal effects of the imidacloprid on the metabolic characteristics based on high-throughput non-targeted metabolomics in *Aphis gossypii* Glover. Ecotoxicol. Environ. Saf..

[47] Ren H., Pu Q., Yang X., Kashyap S., Liu S. (2025). Regulatory mechanisms of nitrogen homeostasis in insect growth and development. Insect Sci..

[48] Yousuf P.Y., Shabir P.A., Hakeem K.R. (2021). miRNAomic approach to plant nitrogen starvation. Int. J. Genom..

[49] Ren X., Guo R., Akami M., Niu C. (2022). Nitrogen acquisition strategies mediated by insect symbionts: A review of their mechanisms, methodologies, and case studies. Insects.

[50] Aqueel M.A., Raza A.B.M., Balal R.M., Shahid M.A., Mustafa I., Javaid M.M., Leather S.R. (2015). Tritrophic interactions between parasitoids and cereal aphids are mediated by nitrogen fertilizer. Insect Sci..

[51] Isoe J., Petchampai N., Isoe Y.E., Co K., Mazzalupo S., Scaraffia P.Y. (2017). Xanthine dehydrogenase-1 silencing in *Aedes aegypti* mosquitoes promotes a blood feeding–induced adulticidal activity. FASEB J..

[52] Li Z., Zhao M., Li L., Yuan Y.Y., Chen F.J., Parajulee M.N., Ge F. (2023). Azotobacter inoculation can enhance the resistance of Bt cotton to cotton bollworm, *Helicoverpa armigera*. Insect Sci..

[53] Jones T.B., Mackey T., Juba A.N., Amin K., Atyam A., McDole M., Yancy J., Thomas T.C., Buhlman L.M. (2024). Mild traumatic brain injury in *Drosophila melanogaster* alters reactive oxygen and nitrogen species in a sex-dependent manner. Exp. Neurol..

[54] Bar-Even A., Flamholz A., Noor E., Milo R. (2012). Rethinking glycolysis: On the biochemical logic of metabolic pathways. Nat. Chem. Biol..

[55] Ankrah N.Y., Wilkes R.A., Zhang F.Q., Zhu D., Kaweesi T., Aristilde L., Douglas A.E. (2020). Syntrophic splitting of central carbon metabolism in host cells bearing functionally different symbiotic bacteria. ISME J..

[56] Sadekuzzaman M.D., Gautam N., Kim Y. (2017). A novel calcium-independent phospholipase A2 and its physiological roles in development and immunity of a lepidopteran insect, *Spodoptera exigua*. Dev. Comp. Immunol..

[57] Rajam M.V. (1991). Insecticidal activity of inhibitors of polyamine biosynthesis on *Spodoptera litura* F. larvae. Indian J. Exp. Biol..

[58] Zawisza-Raszka A., Dolezych B. (2008). Acetylcholinesterase, catalase and glutathione S-transferase activity in beet armyworm (*Spodoptera exigua*) exposed to nickel and/or diazinon. Acta Biol. Hung..

[59] Hu B., Hu S., Huang H., Wei Q., Ren M., Huang S., Tian X., Su J. (2019). Insecticides induce the co-expression of glutathione S-transferases through ROS/CncC pathway in *Spodoptera exigua*. Pestic. Biochem. Physiol..

[60] Isman M.B. (2006). Botanical insecticides, deterrents, and repellents in modern agriculture and an increasingly regulated world. Annu. Rev. Entomol..

[61] Pavela R. (2016). History, presence and perspective of using plant extracts as commercial botanical insecticides and farm products for protection against insects–a review. Plant Prot. Sci..

[62] Chen C., Cai N., Chen J., Wan C. (2019). Clove essential oil as an alternative approach to control postharvest blue mold caused by Penicillium italicum in citrus fruit. Biomolecules.

[63] Čolović M., Lazarević-Pašti T., Vasić V., Mayes C. (2015). Toxic effects of chlorpyrifos and its metabolites on some physiologically important enzymes: ATPases, cholinesterases, peroxidases. Biochemistry Research Trends. Chlorpyrifos. Toxicological Properties, Uses and Effects on Human Health and the Environment.

[64] Hou W., Jiang C., Zhou X., Qian K., Wang L., Shen Y., Zhao Y. (2016). Increased expression of P-glycoprotein is associated with chlorpyrifos resistance in the German cockroach (Blattodea: Blattellidae). J. Econ. Entomol..

[65] Chen Y., Zhang C., Li W., Lan R., Chen R., Hu J., Yang C., Wang P., Tang B., Wang S. (2024). Residues of chlorpyrifos in the environment induce resistance in *Aedes albopictus* by affecting its olfactory system and neurotoxicity. Sci. Total Environ..

[66] Cui S., Wang L., Qiu J., Liu Z., Geng X. (2017). Comparative metabolomics analysis of *Callosobruchus chinensis* larvae under hypoxia, hypoxia/hypercapnia and normoxia. Pest Manag. Sci..

[67] Williamson S.M., Moffat C., Gomersall M.A., Saranzewa N., Connolly C.N., Wright G.A. (2013). Exposure to acetylcholinesterase inhibitors alters the physiology and motor function of honeybees. Front. Physiol..

[68] Brinzer R.A., Henderson L., Marchiondo A.A., Woods D.J., Davies S.A., Dow J.A. (2015). Metabolomic profiling of permethrin-treated *Drosophila melanogaster* identifies a role for tryptophan catabolism in insecticide survival. Insect Biochem. Mol. Biol..

[69] Cox J.E., Thummel C.S., Tennessen J.M. (2017). Metabolomic studies in *Drosophila*. Genetics.

[70] Vasamsetti B.M.K., Kim J., Chon K., Kim B.S., Yoon C.Y., Hwang S., Park K.H. (2024). Molecular impact of sublethal spinetoram exposure on honeybee (*Apis mellifera*) larval and adult transcriptomes. Int. J. Mol. Sci..

[71] Adams R.P. (2007). Identification of Essential Oil Components by Gas Chromatography/Mass Spectrometry.

[72] Mondello L. (2015). Mass Spectra of Flavors and Fragrances of Natural and Synthetic Compounds.

[73] NIST23 (National Institute of Standards and Technology) (2023). NIST/EPA/NIH Mass Spectral Database.

[74] Kar S., Nayak R.N., Sahoo N.R., Bakhara C.K., Panda M.K., Pal U.S., Bal L.M. (2021). Rice weevil management through application of silica nano particle and physico-chemical and cooking characterization of the treated rice. J. Stored Prod. Res..

[75] Kavallieratos N.G., Athanassiou C.G., Peteinatos G.G., Boukouvala M.C., Benelli G. (2018). Insecticidal effect and impact of fitness of three diatomaceous earths on different maize hybrids for the eco-friendly control of the invasive stored-product pest *Prostephanus truncatus* (Horn). Environ. Sci. Pollut. Res..

[76] Kavallieratos N.G., Eleftheriadou N., Boukouvala M.C., Skourti A., Filintas C.S., Gidari D.L.S., Maggi F., Rossi P., Drenaggi E., Morshedloo M.R. (2024). Exploring the efficacy of four Apiaceae essential oils against nine stored-product pests in wheat protection. Plants.

[77] Misra B. (2019). Steps for Building an Open Source EI-MS Mass Spectral Library for GC-MS -Based Metabolomics. https://www.protocols.io/view/steps-for-building-an-open-source-ei-ms-mass-spect-eq2ly33rqgx9/v1.

[78] Sumner L.W., Amberg A., Barrett D., Beale M.H., Beger R., Daykin C.A., Fan W.M.T., Fiehn O., Goodacre R., Griffin J.L. (2007). Proposed minimum reporting standards for chemical analysis: Chemical analysis working group (CAWG) metabolomics standards initiative (MSI). Metabolomics.

[79] Zar J.H. (2014). Biostatistical Analysis.

[80] Scheff D.S., Arthur F.H. (2018). Fecundity of *Tribolium castaneum* and *Tribolium confusum* adults after exposure to deltamethrin packaging. J. Pest Sci..

[81] Sall J., Lehman A., Creighton L. (2001). JMP Start Statistics. A Guide to Statistics and Data Analysis Using JMP and JMP IN Software.

[82] SAS Institute Inc (2021). Using JMP 16.2.

[83] Sokal R.R., Rohlf F.J. (1995). Biometry: The Principles and Practice of Statistics in Biological Research.

[84] Snedecor G.W., Cochran W.G. (1989). Statistical Methods.

